# Minimally invasive plate osteosynthesis using the oblong hole of a locking plate for comminuted distal fibular fractures

**DOI:** 10.1186/s13018-021-02441-2

**Published:** 2021-04-27

**Authors:** Young Uk Park, Sung Jae Kim, Hyong Nyun Kim

**Affiliations:** 1 Department of Orthopedic Surgery, Ajou University Hospital, Ajou University School of Medicine, Suwon, Gyeonggi-do Republic of Korea; 2 Department of Orthopedic Surgery, Dongtan Sacred Hospital, Hallym University, Hwaseong, South Korea; 3 Department of Orthopedic Surgery, Kangnam Sacred Heart Hospital, Hallym University College of Medicine, 948-1, Dalim-1dong, Youngdeungpo-gu, Seoul, 150-950 Republic of Korea

**Keywords:** Fibular fracture, Comminution, Locking plate, Minimally invasive plate osteosynthesis

## Abstract

**Background:**

Nonunion is a rare complication for distal fibular fractures. However, when there is a high degree of comminution, nonunion may occur. In this article, we describe a novel technique that uses the oblong hole of a locking plate to lengthen the fibula for fracture reduction. This technique is straightforward and allows for easy control of the comminuted fracture to restore length and rotation at the time of plate application without opening the fracture site.

**Methods:**

Thirty-five consecutive patients, who were treated with the minimally invasive plate osteosynthesis (MIPO) technique for comminuted distal fibular fractures were retrospectively studied. The study included 19 men and 16 women, with a mean age of 47.0 years (range, 20 to 72). There were 3 lateral malleolar fractures with deltoid injury, 11 bimalleolar fractures, 7 trimalleolar fractures, and 14 distal tibiofibular fractures. The quality of fracture reduction was assessed by comparing the radiologic parameters (fibular length, talocrural angle, and medial clear space) between the affected ankle and the contralateral uninjured ankle.

**Results:**

Two patients were not reachable and 5 declined to visit the clinic. For these 7 patients, the latest outcomes that were measured prospectively were used. Postoperative radiographs showed well-aligned ankle mortise, with fibular length restoration. The mean Olerud-Molander ankle score was 82.1 ± 10.7 at a mean of 27.2 months (range, 12 to 58). There was one case of nonunion and one case of superficial peroneal nerve injury.

**Conclusion:**

The MIPO technique, using the oblong hole of a locking plate, achieved satisfactory restoration of length and rotation, bone union, and clinical outcomes for the comminuted distal fibular fractures.

## Background

**Fig. 1:**
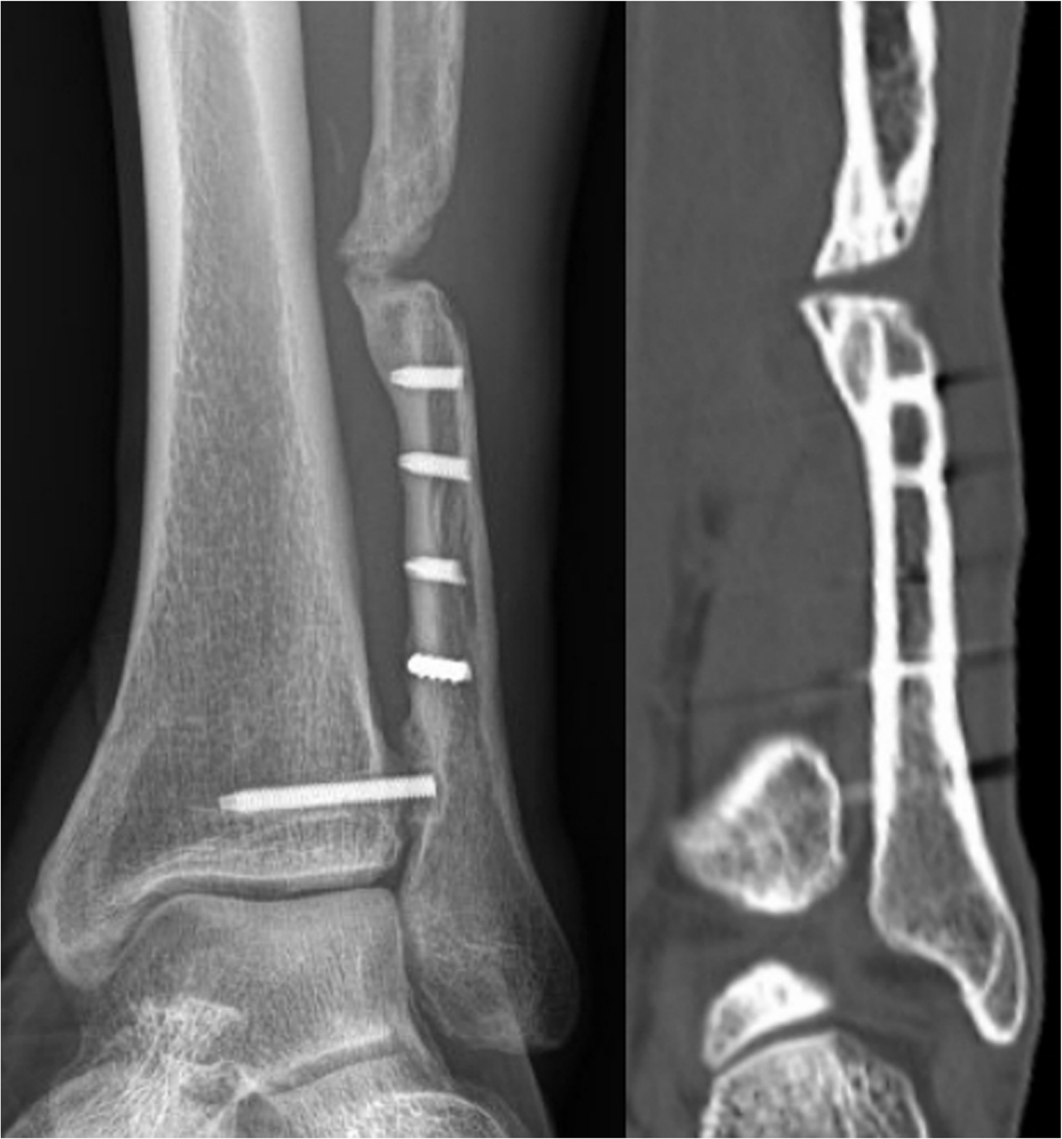
A nonunion is shown after open reduction and internal fixation of a comminuted distal fibular fracture

Ankle fractures are commonly treated by orthopedic surgeons, and clinical results for open reduction and internal fixation (ORIF) of unstable fractures are generally good [1–7]. Nonunion is a rare complication, because the fracture frequently occurs in the cancellous portion of the distal fibula, where it is adequately vascularized by numerous, randomly distributed, metaphyseal arteries that supply that region of the bone. When it occurs more proximally, it is well surrounded by the peroneus brevis muscle to enhance healing. Malrotation and shortening can be more problematic than nonunion. A malunited fracture with fibular shortening and lateral talus shift is indicative of a poor outcome, with pain, swelling, or stiffness often leading to degenerative arthritis [8–10]. This may be the reason why surgeons prefer the open technique, which helps to achieve an anatomic reduction through direct visualization, compared to the minimally invasive technique, which can sometimes result in improper reduction by closed methods [11]. Furthermore, the open technique is much straightforward compared to the minimally invasive technique. However, when there is a high degree of comminution or when the fracture is opened, nonunion may occur (Fig. 1). Standard open approach that violates soft tissue attachments might jeopardize the viability of the comminuted fracture fragments [7, 12–14]. Occasionally, it is difficult to achieve anatomic reduction, even with the open technique, when the fracture is highly comminuted [15]. Fractures with poor skin condition may result in wound problems and infection as a result of using the standard ORIF. Minimally invasive techniques are being used in fractures around the ankle [16–18].

In this article, we describe a novel technique that uses the oblong hole of a locking plate to lengthen the fibula for fracture reduction. This technique is straightforward and allows for easy control of the comminuted fracture to restore length and rotation at the time of plate application without opening the fracture site.

## Materials and methods

### Subjects

Thirty-five consecutive patients, who were treated with the minimally invasive plate osteosynthesis (MIPO) technique for comminuted distal fibular fractures between May 2014 and June 2018, were retrospectively studied. Institutional review board approval was obtained, as well as informed consent from all patients. Indications for the MIPO surgery were distal fibular fractures with comminution or with extensive soft-tissue injury over the fracture. Weber C ankle fractures with comminution, and distal tibiofibular fractures with comminution on the fibula, were indicated for the current technique. Contraindications included Weber A and B fractures, which could be treated with the standard ORIF. Open fractures that required wound debridement were also excluded. Patients’ age, sex, mechanism of injury, concurrent injuries, and comorbidities did not constitute factors for inclusion or exclusion. The study included 19 men and 16 women, with a mean age of 47.0 years (range, 20 to 72). There were 3 lateral malleolar fractures with deltoid injury, 11 bimalleolar fractures, 7 trimalleolar fractures, and 14 distal tibiofibular fractures. According to the Lauge-Hansen classification [19], among 21 ankle fractures, 14 cases were pronation-external rotation fractures and 7 cases were pronation abduction fractures.

### Operative techniques

**Fig. 2:**
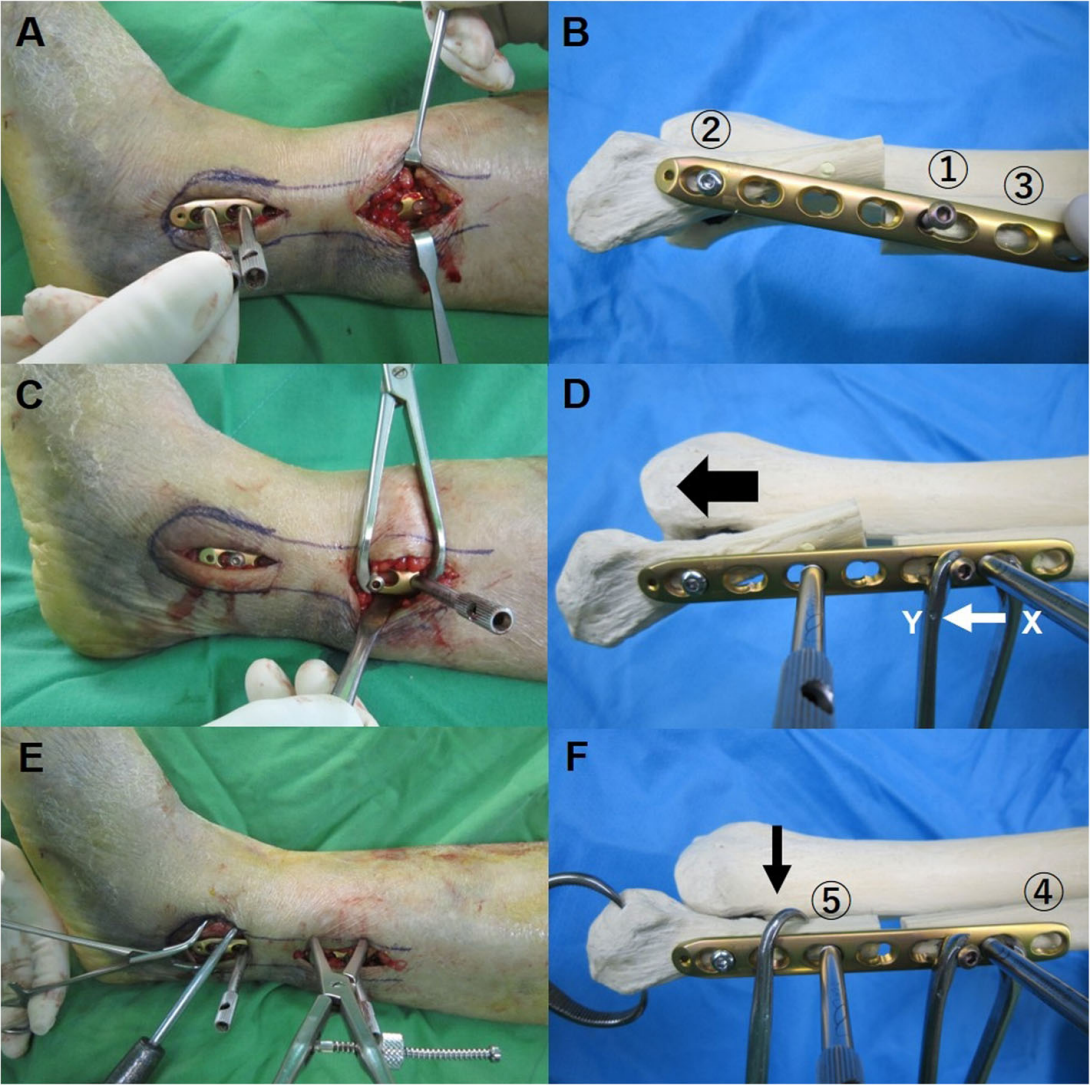
A locking plate with an elongated oblong hole was introduced through a small incision (**a**). One cortical screw was introduced in the most distal hole ② of the plate and inserted into the center of the distal fibula. Another long cortical screw was inserted into the center of the proximal fragment through the elongated hole ① as distally as possible, but not completely against the plate (**b**). A threaded LCP drill sleeve was locked at the second locking hole ③. A bone holding clamp was used to compress the cortical screw and drill sleeve (**c**). One arm of the clamp (Y) held the cortical screw and the other arm (X) pulled down the drill sleeve when the clamp was compressed (white arrow). As the drill sleeve was locked on the plate, and the plate was fixed to the distal fragment, the distal fragment could be pushed downward (black arrow) and the fracture distracted (dotted arrow) when the bone holding clamp was compressed (**d**). Another drill sleeve was locked at the distal locking hole ⑤ to hold the plate and adjust to the longitudinal axis of the proximal fragment. A bone hook was introduced to align the distal fragment, and a reduction clamp was placed on the distal end of the fibula to restore rotation (**e**, **f**). When anatomic reduction with the correct fibular length was achieved, screws were inserted through the holes ④ while the fibular length was maintained with the clamp

**Fig. 3:**
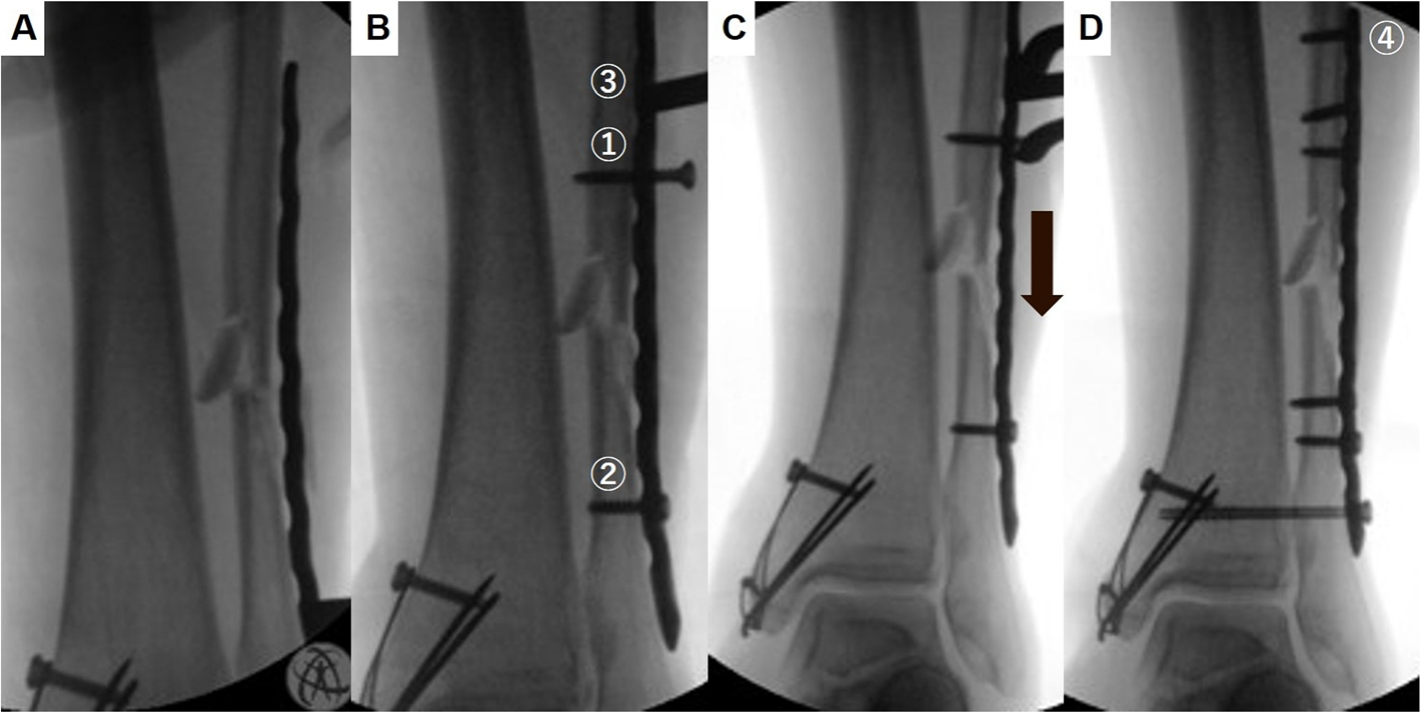
Fluoroscopic images show the technique. After introducing the plate through a small incision (**a**), cortical screws were inserted on the distal hole ② and the elongated hole ① (**b**). A bone holding clamp was used to compress the cortical screw and the drill sleeve locked to the plate ③. When the clamp was compressed, the distal fragment could be pushed downward (black arrow) to restore fibular length (**c**). Screws were inserted through the holes ④ while the reduction was maintained with the clamp (**d**)

Under general or spinal anesthesia, the patient was placed in a supine position with a thigh tourniquet. Radiographs of the contralateral uninjured ankle were taken with the C-arm image intensifier and saved for later use as a reference for anatomic reduction of the fracture. In patients with bimalleolar or trimalleolar fractures, the medial malleolus was first stabilized to allow for comparison with the contralateral uninjured ankle, since symmetry of the clear spaces and other radiologic parameters cannot be assessed without anatomic reduction of the medial malleolus. In patients with distal tibiofibular fractures, who were to be treated with MIPO for both the tibia and fibula, the tibia was fixed first, due to increased difficulty in reduction of the tibia using closed reduction methods after fibular fixation. The correct length of a locking compression plate (LCP), namely, metaphyseal plate 3.5 (Synthes, Solothurn, Switzerland), was chosen under C-arm image intensifier. A small longitudinal incision was made over the lateral malleolus, distal to the comminuted fracture. The metaphyseal end of the LCP metaphyseal plate, which is thinner than the opposite end, was introduced through the incision and advanced proximally over the periosteum and under the skin without removing any fragments (Figs. 2a, 3a). On the metaphyseal end of this plate, the third hole is an elongated combi-hole. The plate was advanced proximally until the distal portion of the elongated hole was positioned about 1 cm proximal to the fracture.

**Fig. 4:**
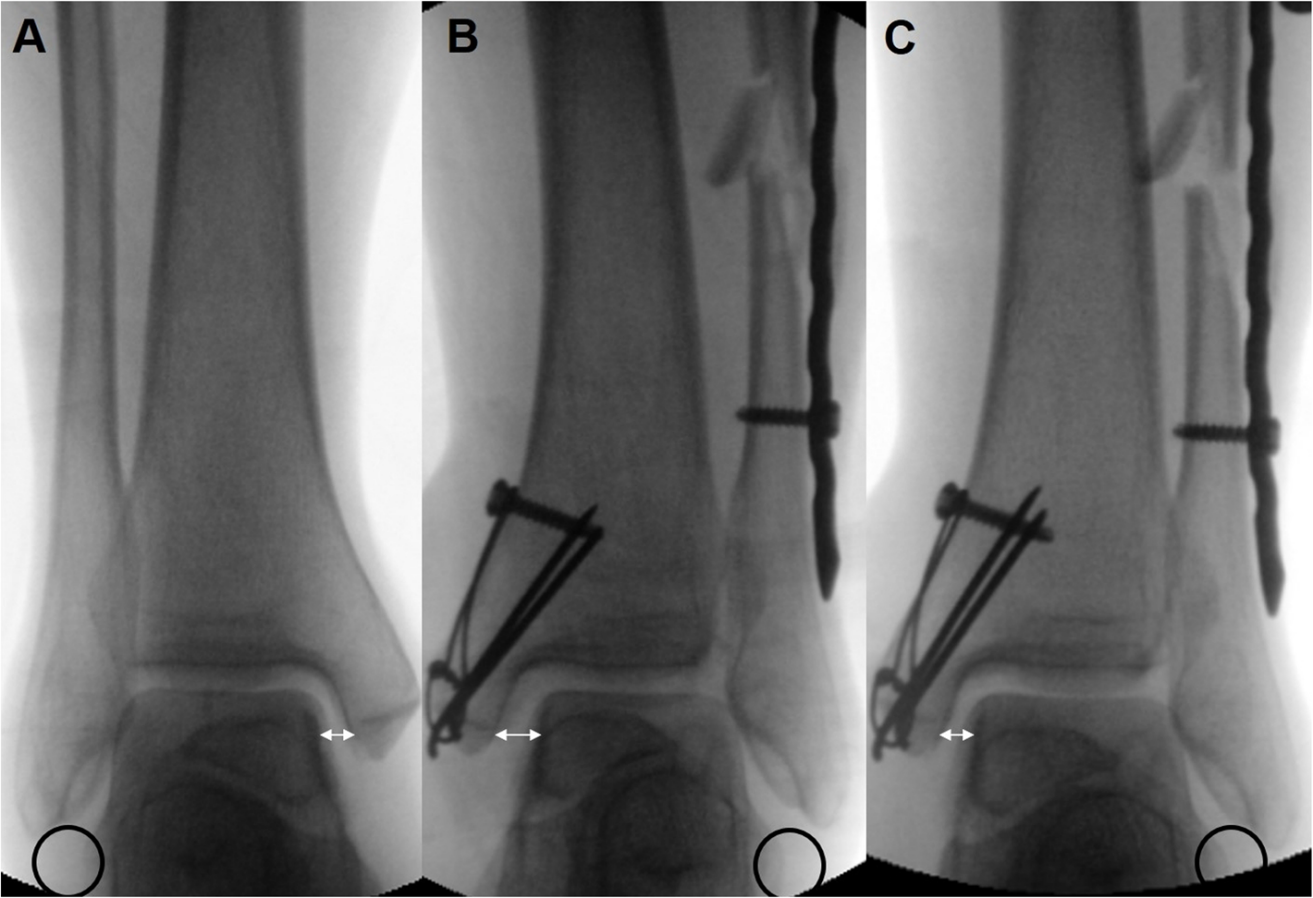
The amount of distraction was determined by comparing a fluoroscopic image of the affected ankle to that of the contralateral uninjured ankle (**a**) based on both the talocrural angle, a perfectly equidistant and parallel joint space, and the contour of the lateral part of the articular talus surface that continues as an unbroken curve to the distal fibular recess. The fluoroscopic image shows fibular shortening and increased width of the medial clear space (**b**) which was restored after using the current technique (**c**)

A second longitudinal incision was made proximal to the fracture, and the plate was visualized in the proximal incision. Great care was taken not to injure the superficial peroneal nerve [7]. The plate was adjusted so that the most distal hole and the elongated hole could be place on the center of the fibula (Fig. 2b). One cortical screw was introduced in the most distal hole of the plate and inserted into the center of the distal fibula to fix the plate to the bone (Fig. 2b). However, the positioning or the fixation of this screw was done in a manner to allow for movement of the distal fibula during posterior fracture reduction. Another long cortical screw was inserted into the center of the proximal fragment through the elongated hole as distally as possible, but not completely against the plate (Figs. 2b, 3b). A threaded LCP drill sleeve was locked at the second proximal locking hole (Figs. 2c, 3b). A bone holding clamp was used to gradually compress the cortical screw and drill sleeve. One arm of the clamp held the cortical screw, which was connected to the proximal fragment, and the other arm pulled down the drill sleeve when the clamp was compressed (Figs. 2d, 3c). As the drill sleeve was locked on the plate, and the plate was fixed to the distal fragment, the distal fragment could be pushed downward and the fracture distracted when the bone holding clamp was compressed (Figs. 2d, 3c). Another drill sleeve was locked at the distal locking hole to hold the plate and adjust to the longitudinal axis of the proximal fragment. A bone hook was introduced to align the distal fragment, and a reduction clamp was placed on the distal end of the fibula to restore rotation (Fig. 2e, f). The amount of distraction was determined by comparing a fluoroscopic image of the affected ankle to that of the contralateral uninjured ankle based on both the talocrural angles, a perfectly equidistant and parallel joint space, and the contour of the lateral part of the articular talus surface that continues as an unbroken curve to the distal fibular recess (Fig. 4). The elongated hole of the metaphyseal LCP 3.5 (Synthes, Solothurn, Switzerland) was approximately 8 mm and this was the largest amount of distraction that was possible using the hole. However, additional 5 mm of distraction was possible using another combi-hole of the plate.

When anatomic reduction with the correct fibular length was achieved, screws were inserted through the holes while the fibular length was maintained with the clamp. Next, the clamp and drill sleeve were removed so that other screws could be inserted (Fig. 3d). A locking plate with different manufacturers could also be used when the plate had an oblong hole or figure-8 hole so that the screw could move longitudinally inside the hole for fibular lengthening. For trimalleolar fractures, reduction of the distal fibular fracture may indirectly reduce the posterior malleolar fragment by ligamentotaxis, thus eliminating the need for extensive exposure and increased soft tissue dissection [20]. Percutaneous screw fixation was performed as described by Lee et al. [20]. Postoperatively, the patients were restricted from weight-bearing for 6 weeks in a posterior splint. When a syndesmotic screw was fixed for syndesmotic injury, weight-bearing was delayed for 8 to 10 weeks. For distal tibiofibular fractures without syndesmotic injury, reduction of the tibia usually reduced the length of the fibular that did not require fibular lengthening. However, using the oblong hole of a locking plate and the present technique allowed for easy control of the comminuted fibular fracture to restore length and rotation at the time of plate application without opening the fracture site. In cases when fracture gap had to be closed, it could be achieved by distracting the drill sleeve locked at the second proximal locking hole proximally from the cortical screw inserted through the oblong hole using a small laminar spreader or a Gelpi retractor. Bone hook could be used to realign the displaced fibula (Fig. 2e, f).

### Postoperative assessment

**Fig. 5:**
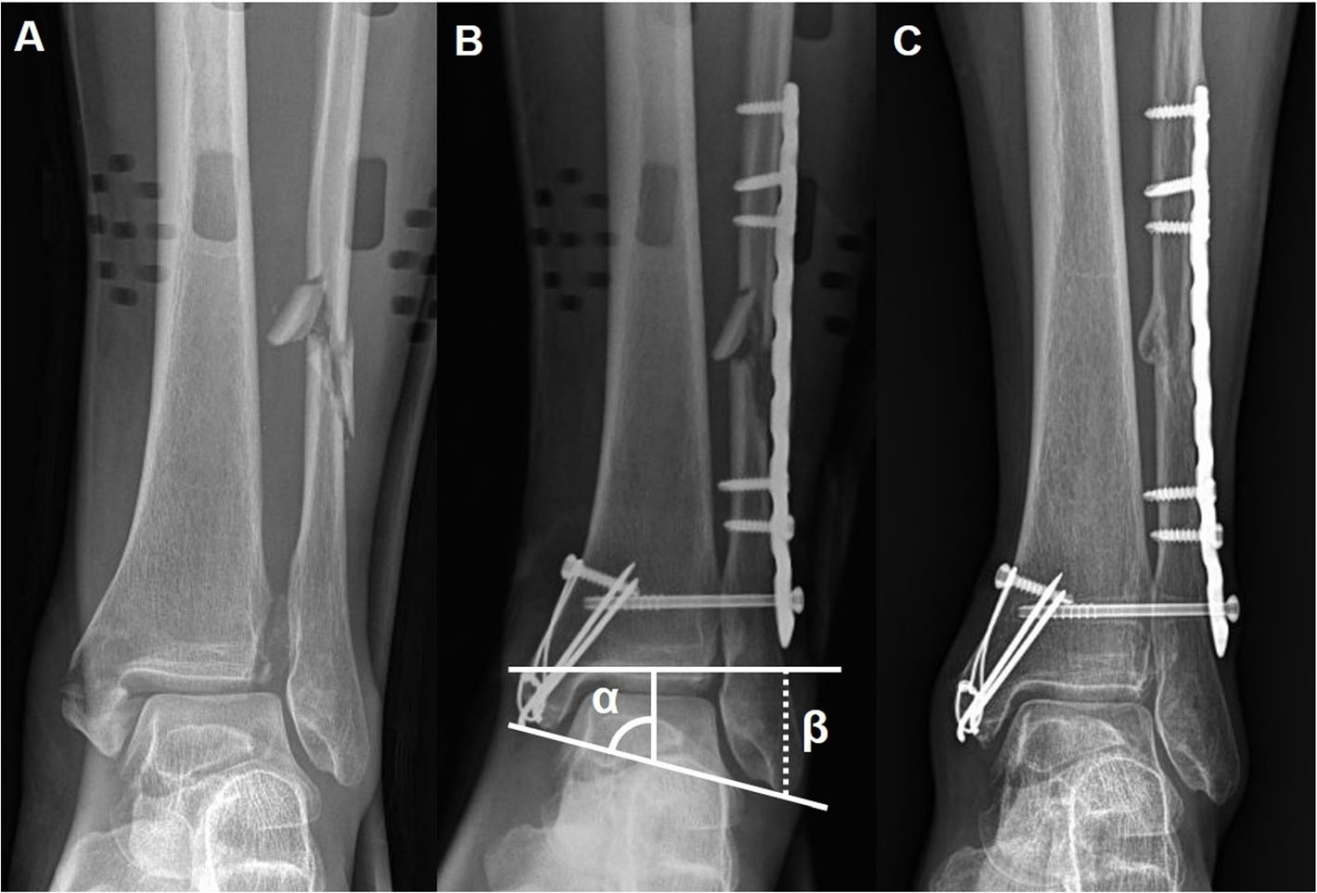
A preoperative radiograph shows a trimalleolar fracture with fibular comminution (**a**). A postoperative radiograph shows restoration of the length and radiologic parameters. Talocrural angle (*α*) was defined as the angle between the distal tibial articular line and the line connecting the tips of the distal fibula and medial malleolus. Fibular length (*β*) was defined as the distance from the distal fibular tip to the distal tibial articular line (**b**). One-year follow-up radiograph shows good union (**c**)

The quality of fracture reduction was assessed by comparing the radiologic parameters (fibular length, talocrural angle, and medial clear space) between the affected ankle and the contralateral uninjured ankle [21, 22]. Fibular length was defined as the distance from the distal fibular tip to the distal tibial articular line. The talocrural angle consists of the angle between the distal tibial articular line and the line connecting the tips of the distal fibula and medial malleolus. The medial clear space consists of the distance between the medial border of the talus and the lateral border of the medial malleolus on a line parallel, and 5 mm below, the talar dome (Fig. 5). The radiologic parameters were measured by a single orthopedic surgeon blinded to the study details. Fracture union was decided when simple radiographs showed at least three of the four cortices bridged by visible callus and on clinical aspects, such as resolution of pain on the fracture site. Clinical outcome scores were assessed with the Olerud-Molander scores (OMS) [23]. The 100-point 9-category OMS is a validated scoring system that includes aspects of outcome that are considered useful: pain, stiffness, swelling, stair climbing, running, jumping, squatting, use of supports and ability to work, and activities of daily living [23]. Patients were followed up for a minimum of 1 year at regular intervals and were invited for a final follow-up office visit for the study.

### Statistical analysis

Data normality was assessed by the Kolmogorov–Smirnov test. Paired *t* test was used to compare the radiologic parameters between the operated ankle and the contralateral normal ankle using the SPSS version 20.0 (SPSS, SPSS Inc., Chicago, IL, USA). Statistical significance was defined at the 5% (*p* < 0.05) level.

## Results

Two patients were not reachable and 5 declined to visit the clinic. For these 7 patients, the latest outcomes that were measured prospectively were used. The mean duration of follow-up was 27.2 months (range, 12 to 58). Final follow-up radiographs showed well-aligned ankle mortise, with fibular length restoration, on the fractured ankle as compared with the contralateral uninjured ankle (Table 1).

**Table 1:** Radiologic outcomes

	Fractured ankle*	Contralateral uninjured ankle*	***P*** value
**Fibular length (mm)**	27.5 ± 3.2	27.3 ± 3.6	0.27
**Talocrural ankle (degrees)**	78.9 ± 2.4	79.2 ± 2.6	0.15
**Medial clear space (mm)**	2.4 ± 0.4	2.5 ± 0.4	0.16

*The values are given as the mean ± standard deviation

**Fig. 6:**
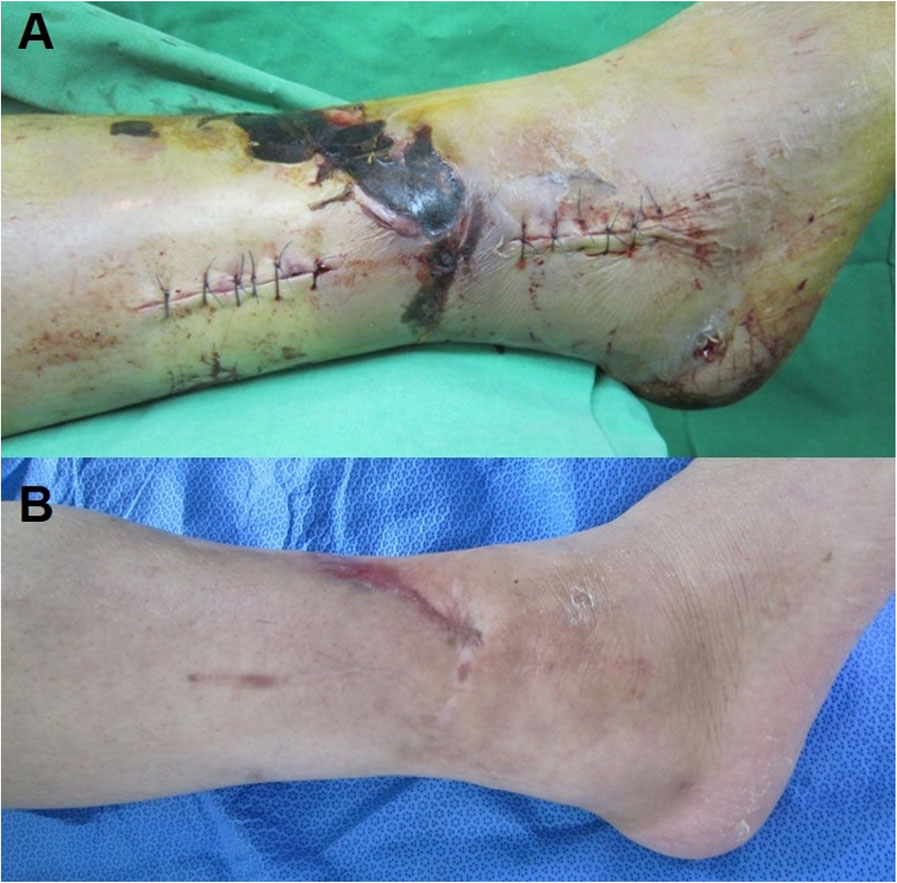
An intraoperative photo shows extensive soft tissue injury around the distal tibiofibular fracture. After applying temporary external fixation for 2 weeks, minimally invasive technique was used (**a**). The wound healed without skin graft or flap surgery and the fracture healed without infection (**b**)

The mean OMS at the final follow-up was 82.1 ± 10.7. Thirty-four fractures healed in an average of 11.4 weeks (range, 7 to 26). There was one case of nonunion and one case of superficial peroneal nerve injury [24]. The case with nonunion was combined with open distal tibial fracture that was treated by ilizarov external fixation. The tibia fracture was converted to intramedullary nailing, and the fibular nonunion was asymptomatic and did not require further surgery. One case with superficial peroneal nerve injury was combined with extensive soft tissue injury (Fig. 6). In this case, the superficial peroneal nerve was suspected to be injured at the time of the fracture.

The datasets used and analyzed during the current study are available from the corresponding author upon request.

## Discussion

The most important finding of the current study was that the MIPO technique using the oblong hole of a locking plate achieved satisfactory restoration of length and rotation, bone union, and clinical outcomes for the comminuted distal fibular fractures.

Several devices have been developed to enable minimally invasive surgery for lateral malleolar fracture [12, 25–30]. Intramedullary nails are reported to be useful and are associated with good clinical outcomes [12, 14, 30, 31]. Indeed, they are especially beneficial in small skin incisions, minimal soft tissue dissection, and less prominent hardware. However, we believe the locking screw and plate system can build stronger construct to maintain the length [32]. Locking plates are commonly used for a “bridge plate” function, preserving periosteal and soft tissue blood supply and providing fixed angle stability. Locked internal fixation, used in conjunction with a minimally invasive approach, minimizes damage and may optimize fracture healing.

The current technique uses a locking plate with a minimally invasive approach to lengthen the fibula and simultaneously stabilize the restored length and rotation. There are several methods to distract the shortened fibula without opening the fracture. An AO distractor can be used, in which a plate is applied to the distal fibular fragment, one arm of the distractor hooks into the proximal end of the plate and the other arm is applied to a temporary screw inserted proximal to the plate for distraction [33, 34]. However, this technique requires proximal extension of the incision and exposure for temporary screw placement. Also, this technique requires a special device (AO distractor) that may not be available in all surgical settings. A pointed reduction clamp can be used to distract the shortened fibula, as well as a K-wire to temporarily maintain the length. This is a useful method, but sometimes the K-wire may block the placement of the plate [13]. In our technique, a plate is applied to lengthen the fibula, allowing for stabilization of the fracture reduction. In 35 consecutive patients who were treated with our technique for comminuted distal fibular fractures, satisfactory restoration of length and rotation was achieved, and all cases achieved bone union, except for one case with open tibial fracture that showed delayed union using an ilizarov external fixator. We suspect that the unstable tibia negatively affected healing of the fibula to result in nonunion.

Minimally invasive techniques are being used in ankle fractures [16–18]. However, there are some limitations of using the MIPO technique for fibular lengthening. For example, distraction is limited to the size of the oblong hole within the plate. However, when using the elongated combi-hole and an adjacent combi-hole within the LCP metaphyseal plate 3.5, a maximum distraction of 13 mm is possible [35]. Even when the fibula is severely shortened, reduction of talus under the tibial plafond can pull, to some extent, the distal portion of fibula by the lateral ligaments. Surgeons can use a reduction clamp to purchase and distract the distal fibula in a closed method while applying the locking plate, and subsequently apply the current MIPO technique to precisely restore the length and rotation under C-arm image intensifier [9].

There are limitations to the study, including its retrospective design without a comparative group. We also recognize that the relatively small cohort and single surgeon experience allow for less generalizability. The quality of fracture reduction and osseous consolidation were not assessed with postoperative computed tomography, which could also constitute as a limitation of the study. Further comparative study, with larger numbers of patients with comminuted distal fibular fractures, is necessary to clarify the advantages and disadvantages of the current MIPO technique.

## Conclusion

The MIPO technique, using the oblong hole of a locking plate, achieved satisfactory restoration of length and rotation, bone union, and clinical outcomes for the comminuted distal fibular fractures.

## Data Availability

The datasets used and analyzed during the current study are available from the corresponding author on reasonable request.

## Abbreviations

MIPO: Minimally invasive plate osteosynthesis
ORIF: Open reduction and internal fixation
LCP: Locking compression plate
OMS: Olerud-Molander scores
